# Medication-Facilitated Advanced Airway Management with First-Line Use of a Supraglottic Device – A One-Year Quality Assurance Review

**DOI:** 10.1017/S1049023X22000802

**Published:** 2022-08

**Authors:** Bethany J. Johnston, Alison K. Leung, Charles W. Hwang, Jason M. Jones, Muhammad Abdul Baker Chowdhury, Alicia Buck, Desmond E. Fitzpatrick, David A. Meurer, Torben K. Becker

**Affiliations:** Department of Emergency Medicine, University of Florida College of Medicine, Gainesville, Florida USA

**Keywords:** airway management, endotracheal intubation, rapid sequence airway, supraglottic device

## Abstract

**Introduction:**

Airway management is a controversial topic in modern Emergency Medical Services (EMS) systems. Among many concerns regarding endotracheal intubation (ETI), unrecognized esophageal intubation and observations of unfavorable neurologic outcomes in some studies raise the question of whether alternative airway techniques should be first-line in EMS airway management protocols. Supraglottic airway devices (SADs) are simpler to use, provide reliable oxygenation and ventilation, and may thus be an alternative first-line airway device for paramedics. In 2019, Alachua County Fire Rescue (ACFR; Alachua, Florida USA) introduced a novel protocol for advanced airway management emphasizing first-line use of a second-generation SAD (i-gel) for patients requiring medication-facilitated airway management (referred to as “rapid sequence airway” [RSA] protocol).

**Study Objective:**

This was a one-year quality assurance review of care provided under the RSA protocol looking at compliance and first-pass success rate of first-line SAD use.

**Methods:**

Records were obtained from the agency’s electronic medical record (EMR), searching for the use of the RSA protocol, advanced airway devices, or either ketamine or rocuronium. If available, hospital follow-up data regarding patient condition and emergency department (ED) airway exchange were obtained.

**Results:**

During the first year, 33 advanced airway attempts were made under the protocol by 23 paramedics. Overall, compliance with the airway device sequence as specified in the protocol was 72.7%. When ETI was non-compliantly used as first-line airway device, the first-pass success rate was 44.4% compared to 87.5% with adherence to first-line SAD use. All prehospital SADs were exchanged in the ED in a delayed fashion and almost exclusively per physician preference alone. In no case was the SAD exchanged for suspected dislodgement evidenced by lack of capnography.

**Conclusion:**

First-line use of a SAD was associated with a high first-pass attempt success rate in a real-life cohort of prehospital advanced airway encounters. No SAD required emergent exchange upon hospital arrival.

## Introduction

Airway management is a crucial component of prehospital medical care. Nonetheless, the use of advanced airway techniques such as endotracheal intubation (ETI) has been controversial almost as long as modern Emergency Medical Services (EMS) systems have existed.^1–3^ Concerns include the risk of unrecognized esophageal intubation, ability to achieve proficiency during paramedic training, skill maintenance in light of the relative scarcity of the procedure, prolonged procedure times, and interruptions in chest compressions during cardiopulmonary resuscitation that may occur during ETI.^1,2^ Additionally, studies comparing ETI with more basic airway maneuvers such as bag-valve-mask (BVM) in cardiac arrest have raised concerns that ETI use may be associated with worse neurological outcomes.^3–9^ In one such study of over 600,000 patients with out-of-hospital cardiac arrest (OHCA), 1.0% of patients had favorable neurological outcomes with ETI versus 2.9% of patients in the BVM group.^4^ Endotracheal intubation has been associated with more complications in the pediatric population, including decreased survival (14% versus 33% with BVM) and decreased rate of favorable neurological outcome (8% versus 26%).^8^

With the arrival of supraglottic airway devices (SADs) such as the laryngeal mask airway (LMA) in 1981, clinicians gained access to a device that could quickly provide oxygenation and ventilation along with some protection against aspiration.^10^ Supraglottic airway devices are simpler and faster to insert and provide more expedient and reliable oxygenation and ventilation than BVM or ETI.^11,12^ Additionally, skill retention has proved more enduring with supraglottic devices versus ETI.^13^

Airway management in the emergency setting also frequently requires sedation, most commonly performed by rapid sequence intubation (RSI), in which a sedative and paralytic are simultaneously administered, but these methods put patients at risk for hypoxia and acidosis during the apneic period. Additionally, traditionally administered sedatives such as midazolam and fentanyl can further cause patient harm by inducing hypotension.^14,15^ The concept of delayed sequence intubation using ketamine to preserve respiratory drive has been demonstrated to be effective to facilitate airway management.^14^

Based on this evidence, in 2019, Alachua County Fire Rescue (ACFR; Alachua, Florida USA) introduced a modified protocol for advanced airway management that emphasized the first-line use of a second-generation SAD instead of ETI for patients requiring medication-facilitated airway management with the use of ketamine for sedation. This paper presents the results of a one-year quality assurance review of real-world implementation of this novel protocol.

## Methods

Prior to 2019, only critical care paramedics with advanced training at ACFR were authorized to perform medication-facilitated advanced airway management, including RSI. The medical directors set out to develop and implement a new approach to prehospital airway management using the Plan-Do-Study-Act principles of quality improvement projects (plan: protocol development and training; do: protocol implementation; study: analysis of prehospital airway management under the new protocol; and act: modifications of the protocol). The goal was to improve first-pass success and patient outcome and to expand the available methods of airway management to transport paramedics. Prior to implementation of the protocol, paramedic airway management was limited to the King Tube SAD, BVM, or continuous positive airway pressure (CPAP) with midazolam for sedation when needed. Under the new protocol entitled the rapid sequence airway (RSA) protocol, those paramedics with a primary assignment on a transport ambulance and who received in-person training with the medical directors were cleared for medication-facilitated advanced airway management with primary use of a second-generation SAD (i-gel; Intersurgical; Wokingham, United Kingdom). Under the protocol, the paramedic administers 1-2mg/kg of ketamine intravenous (IV) for sedation, followed by up to two attempts at placing the SAD. If these attempts fail, the paramedic can administer 1.5mg/kg of rocuronium IV followed by a third attempt using the SAD. Finally, if the airway is unable to be secured after paralysis and oxygenation/ventilation is insufficient with BVM, the paramedic can proceed with ETI (or surgical airway, depending on patient stability) to secure the airway. An exception for primary ETI was made for cases of significant burns, severe anaphylaxis, or trauma to the face/neck where concern for airway edema or bleeding above the vocal cords justified the primary use of ETI. All airway attempts required end-tidal carbon dioxide (ETCO_2_) tracings for confirmation. A failed attempt was defined as an attempt resulting in difficulty oxygenating or ventilating, significant air leak, dyssynchrony with ventilation attempts, or lack of appropriate ETCO_2_ tracing. Ketamine was to be re-dosed every 15 minutes for continued sedation at 0.5mg/kg IV. Ventilation continued during transport with the use of a BVM; use of positive end-expiratory pressure was left to provider discretion. Patients in cardiac arrest do not require medication-facilitated airway management and the protocol thus did not apply to them.

One year after implementing the protocol, a quality assurance review at the medical directors’ level was performed to evaluate outcomes and assess real-world compliance with the step-wise approach outlined in the protocol. This quality assurance review was part of the standard quality assurance review. Patients who received airway intervention while in active cardiac arrest were excluded as they did not require any medications for induction or paralysis. To obtain data, the agency’s electronic medical record (EMR) system was queried, searching for the use of the RSA protocol, use of an advanced airway device, or use of either rocuronium or ketamine. Data points extracted included patient age, gender, comorbidities, type of call including chief complaint, indication for airway intervention, advanced airway device and number of attempts, medications and dosages, total scene time, presence or absence of oxygen desaturation, presence or absence of hypotension, airway adjuncts used, and need for vasopressors. Mild hypoxia was defined as an oxygen saturation of less than 90% and severe hypoxia was defined as an oxygen saturation of less than 80%. Mild hypotension was defined as less than 90mmHg systolic and severe hypotension was defined as less than 80mmHg systolic. For patients transported to the hospital that the agency medical directors are employed at, the hospital EMR was accessed to obtain information on the patient’s clinical course after transition of care to the emergency department (ED) team. Access to EMRs at the other receiving facility within the county was not available. Data points extracted included indication for device exchange, type of device and method used, patient length-of-stay in the ED/intensive care unit (ICU)/total hospitalization, admission and discharge diagnosis, patient survival to hospital admission, and patient survival to discharge.

After reviewing and summarizing the raw data using frequentist statistics, a Chi-square analysis was performed to evaluate for an association between the number of airway attempts and use of paralytic or sedative, as well as the presence of hypoxia and hypotension.

The institutional process for approval as a quality improvement project was followed, leading to registration as a quality project exempt from review as human subjects research by the Institutional Review Board. The decision to formally write up the results and analysis was made post-hoc.

## Results

During the first year of the protocol implementation, advanced airway interventions were performed by a cohort of 23 out of 52 paramedics trained.

A total of 33 patients had an airway management attempt made under the RSA protocol (Table 1). Most patients were adult males with multiple comorbidities, most commonly hypertension (33.3%), diabetes (21.2%), and chronic lung disease (21.2%). A small proportion of patients were residents of long-term care or skilled nursing facilities (6.1%), and 6.1% of patients were considered morbidly obese. The vast majority of calls requiring RSA were medical (75.8%), and the most frequently encountered dispatch complaints were “breathing problem” and “unconscious/fainting” (21.2% each). For each encounter, multiple RSA indications could be selected simultaneously (Table 2). Pre-RSA hypoxia and hypotension were present in the majority of patients. Push-dose epinephrine was rarely utilized in these cases; most personnel opted for IV fluid resuscitation alone.

**Table 1.** Patient and Call Characteristics

Patient Characteristics	n (%)
Gender	
Male	21 (63.6)
Female	12 (36.4)
Median Age	65
Comorbidities	
Hypertension	11 (33.3)
Diabetes	7 (21.2)
COPD	7 (21.2)
CAD	3 (9.1)
CHF	3 (9.1)
Morbid Obesity	2 (6.1)
SNF/LTAC Resident	2 (6.1)
CKD	1 (3.0)
Call Type	
Medical	25 (75.8)
Trauma	8 (24.2)
Dispatch Complaint^a^	
Breathing Problem	7 (21.2)
Unconscious/Fainting	7 (21.2)
Cardiac Arrest	4 (12.1)
Traffic Accident	4 (12.1)
Sick Person	2 (6.1)
Stroke/CVA	2 (6.1)

Abbreviations: CAD, coronary artery disease; CHF, congestive heart failure; CKD, chronic kidney disease; COPD, chronic obstructive pulmonary disease; CVA, cerebrovascular accident; GSW, gunshot wound; LTAC, long-term acute care; SNF, skilled nursing facility.

a Altered Mental Status, Burns/Explosion, Hemorrhage/Laceration, Psychiatric Problem, Stab/GSW/Penetrating Trauma, Transfer/Inter-Facility/Palliative Care, and Traumatic Injury had frequency of one which corresponds to 3.0% of the total sample.

**Table 2.** Advanced Airway Indications, Pre-Existing Hypoxia and Hypotension, and Use of Corrective Measures Prior to RSA

Pre-RSA Conditions	n (%)
Indications for Advanced Airway^a^	
Airway Protection	28 (84.9)
Hypoxia	23 (69.7)
Agitation	1 (3.0)
Hypercapnia	1 (3.0)
Pre-RSA Hypoxia	
Severe (SpO_2_ < 80%)	16 (48.5)
Mild (SpO_2_ 80-90%)	8 (24.2)
None	9 (27.3)
Preoxygenation Adjunct Device Used^b^	
No Preoxygenation	0 (0.0)
BVM	28 (84.9)
OPA/NPA	15 (45.5)
NRB	12 (36.4)
CPAP/BIPAP	4 (12.1)
NC	2 (6.1)
Pre-RSA Hypotension	
Severe (Systolic < 80 mmHg)	11 (33.3)
Mild (Systolic 80-90 mmHg)	2 (6.1)
None	19 (57.6)
No Data	1 (3.0)
Pre-RSA Hypotension Intervention	
Intravenous Fluids	12 (92.3)
Push-Dose Epinephrine	1 (7.7)
Vasopressor Infusion	0 (0.0)

Abbreviations: BIPAP, bilevel positive airway pressure; BVM, bag-valve-mask ventilation; CPAP, continuous positive airway pressure; NC, nasal cannula; NPA, nasopharyngeal airway; NRB, nonrebreather mask; OPA, oropharyngeal airway; RSA, rapid sequence airway.

a Multiple indications for advanced airway existed; therefore, percentages total more than 100%.

b Multiple interventions were performed; therefore, percentages total more than 100%.

Most airway attempts were made after sedative induction with ketamine, in compliance with the protocol (Table 3). Approximately one-half of patients received rocuronium. After airway confirmation, re-dosing sedatives during transport was rare. Hypotension and initial hypoxia were common after airway management. Vasopressors were administered in 24% of cases. In the majority of protocol-compliant cases, paramedics achieved adequate ventilation and oxygenation after one attempt with an SAD as confirmed via continuous waveform capnography. Only three cases required two SAD attempts; in no case did an SAD fail or require more than two attempts, precluding the escalation to an endotracheal tube (ETT) or surgical airway. In nine cases (27.3%), contrary to protocol, ETT was selected as the first-line airway device. Only four succeeded in a secure airway after one attempt, yielding first-pass success rate of 44.4% compared to an 87.5% first-pass success rate when the SAD was used.

**Table 3.** Airway Attempt Characteristics

Airway Attempt Characteristics	n (%)
Number of Attempts	
1	25 (75.8)
≥ 2	8 (24.2)
Elapsed Time until Securing Airway (After First Attempt)	
< 1 Minute	23 (69.7)
≥ 1 Minute	10 (30.3)
Airway Confirmation^a^	
End-Tidal Waveform Capnography	32 (97.0)
Auscultation	13 (39.4)
Chest Rise	14 (42.4)
Direct Visualization	7 (21.2)
Induction Agent	
Ketamine	26 (78.8)
None	7 (21.2)
Propofol, Midazolam, Lorazepam, Fentanyl	0 (0.0)
Paralytic Agent	
Rocuronium	18 (54.6)
None	15 (45.4)
Succinylcholine	0 (0.0)
Post-Airway Sedation	
None	27 (81.8)
Ketamine	5 (15.2)
Midazolam	2 (6.0)
Propofol, Lorazepam, or Fentanyl	0 (0.0)
Progression to Secured Airway	
i-gel after 1 Attempt	20 (60.6)
i-gel after 2 Attempts	3 (9.1)
i-gel after 1 Attempt, No ETCO_2_ Attempted for Confirmation	1 (3.0)
ETT after 1 Attempt	4 (12.1)
ETT after 2 Attempts	1 (3.0)
ETT First à i-gel Final	1 (3.0)
ETT First à i-gel à ETT Final	1 (3.0)
ETT First à i-gel à BVM into ED	1 (3.0)
ETT First à BVM into ED	1 (3.0)
i-gel First-Pass Success Rate: 83.3% ETT First-Pass Success Rate: 44.4%	
Complications	
Hypoxia	
Severe (SpO_2_ < 80%)	5 (15.2)
Mild (SpO_2_ 80-90%)	7 (21.2)
None	20 (60.6)
No Data	1 (3.0)
Hypotension	
Severe (Systolic < 80 mmHg)	2 (6.1)
Mild (Systolic 80-90 mmHg)	8 (24.2)
None	22 (66.7)
No Data	1 (3.0)

Note: Number of attempts, time to secure airway, induction agents and paralytics used, need for continued sedation, complications, and progression to secured airway.

Abbreviations: BVM, bag-valve-mask ventilation; ED, emergency department; ETCO_2_, end-tidal CO_2_ capnography; ETT, endotracheal tube.

a Multiple airway confirmation methods were performed; therefore, percentages total more than 100%.

The number of attempts made was not associated with the use of ketamine or rocuronium, nor was the occurrence of post-RSA hypotension or hypoxia. Of cases with hospital records available (n = 20), all patients arriving to the ED with an SAD had the device exchanged, usually via video laryngoscopy (73.3%); no patient required a surgical airway. In all but one hypoxic case, physicians exchanged the SAD to ETT per their preference alone, and in no case was an SAD exchanged for suspected dislodgement due to lack of capnography reading. All patients survived to hospital admission, and 65% survived to hospital discharge. In one case, the prehospital SAD was removed in the ED per patient and family wishes; the patient was discharged to hospice. The two most common diagnoses at hospital discharge were traumatic brain injury (30.4%) and acute hypoxic respiratory failure (26.1%). In-hospital mortality was 34.8%.

## Discussion

Second-generation SADs are recognized as an effective option for prehospital airway management and may reduce complications associated with ETI such as procedural failure and delay in oxygenation. The approach of combining the benefits of medication-facilitated airway management with the advantages of SAD over ETT was first coined RSA in 2007 but is yet to see wide-spread adoption.^16^ In this system, prioritizing SAD as first-line during prehospital medication-facilitated airway management resulted in a very high first-pass and overall success rate. All cases of SAD placement were successful within two attempts.

The SAD was selected as the primary RSA protocol device for several reasons. The SAD is a second-generation SAD featuring a thermoplastic elastomer cuff that conforms to the airway without the need for an inflatable cuff. The lack of a cuff makes the device simpler to insert and eliminates potential complications from over-inflation such as tissue compression.^17^ Several studies have shown that the SAD is quick to insert and is effective as an airway management device across a wide variety of different prehospital airway encounters.^18–20^ The manufacturer indicates that the device can be in place for up to four hours, well exceeding most EMS transport times.^21^

In this protocol, a paralytic (rocuronium) was intended to be delivered only when two post-induction attempts at placement were unsuccessful. The goal was to avoid prolonged paralysis, when possible, to allow for better monitoring of patient sedation and to limit apneic periods, which can be extensive when a paralytic is used. Succinylcholine could have also limited apneic periods, but this was not practical due to lack of refrigeration availability at the time. Ketamine was selected as the sedative of choice due to its ability to preserve respiratory drive and its effects on hemodynamics. Despite the protocol design, in approximately one-half of cases, the paramedic delivered the induction agent and paralytic before the first airway attempt. Neither approach was associated with a significantly increased rate of complications, suggesting that either approach may be appropriate.

A major concern with first-generation SADs is the risk of aspiration. While this review could not collect data on aspiration cohort, previously published data showed no difference between SAD and ETT in a cohort of patients receiving prehospital airway management.^22^

In approximately one-quarter of cases, a paramedic selected ETI as the first-line over SAD, despite an absolute indication being absent, in violation of the protocol. As this was the first year of protocol implementation, this is not entirely unexpected. First-pass success rate with this strategy was only 44% compared to 87% for SAD. Although protocol deviation might have been due to an unknown factor, this review could not identify any patient-specific causes other than paramedic preference (in violation of protocol). Each case of protocol deviation was addressed with the paramedic in a one-on-one meeting with the medical directors. A recent study by Braude, et al found a similar first-pass success rate with different SADs across several agencies. The Agency for Healthcare Research and Quality (Rockville, Maryland USA) recently published a systematic review on prehospital airway management and concluded that first-pass success rates were higher with SADs, supporting this study’s approach and findings.^12,23^

The significant difference in first-pass success stresses the importance of EMS agencies and medical directors in monitoring rates of protocol compliance and device-specific success closely.

Additionally, despite a specific educational focus on re-dosing sedation, ACFR’s paramedics rarely provided more than one dose of sedative during transport. However, transport times in the county are short (average of 14:55 minutes). Nonetheless, the surveillance of re-dosing sedative and analgesic medications in cases where a long-acting paralytic has been delivered is critical, especially in systems with transport times longer than in this county.

Previous large studies comparing ETT with SADs and BVM had looked at outcomes in OHCA which demonstrated either equivalent or better outcomes with SAD and/or BVM use. Current findings are unique in that the protocol applied to non-OHCA patients, but the high first-pass success rate is consistent with data previously presented for the use of SADs in OHCA.^3,9^

As a result of these findings, several additional changes have been made to the protocol. First, education was provided to paramedics on the findings of this analysis and the excellent success rate with use of the SAD. Second, education on airway management and the RSA protocol is provided at least annually to all paramedics. Third, further emphasis has been placed on the need of initial and continued sedation of patients receiving advanced airway management in paramedic airway training. Use of rocuronium has been transitioned to succinylcholine as preferred paralytic to avoid any situation in which a patient could remain paralyzed without adequate sedation now that refrigeration has since become available on all ambulances. Based on this change, first-line use of the paralytic medication along with the sedative is now allowed.

## Limitations

This review reflects data from a single EMS agency with a relatively small number of patient cases in whom the protocol applied. Nonetheless, other published RSA data over multiple years and agencies report similarly small case numbers, stressing that advanced airway management remains a somewhat rare procedure in the prehospital setting.^12^ Additionally, outcome data were only able to be obtained from one of two receiving hospitals. The current EMS system had previously only allowed critical care paramedics to perform drug-facilitated advanced airway management. Consequently, these findings may not be generalizable to all EMS systems or settings.

## Conclusions

One year after the introduction of an RSA protocol that prioritized placement of the SAD facilitated by ketamine, real-world use reflects a high first-pass and overall success rate when care was provided in compliance with the protocol. Hypotension was frequently seen before and after airway management. When ETT was chosen in violation of the protocol, first-pass success rates were low.

## References

[1] Pepe PE , Roppolo LP , Fowler RL. Prehospital endotracheal intubation: elemental or detrimental? Crit Care. 2015;19(1):121. 25887350 10.1186/s13054-015-0808-x PMC4440604

[2] Prekker ME , Kwok H , Shin J , Carlbom D , Grabinsky A , Rea TD. The process of prehospital airway management: challenges and solutions during paramedic endotracheal intubation. Crit Care Med. 2014;42(6):1372–1378. 24589641 10.1097/CCM.0000000000000213 PMC4902016

[3] Benger JR , Kirby K , Black S , et al. Effect of a strategy of a supraglottic airway device vs tracheal intubation during out-of-hospital cardiac arrest on functional outcome the AIRWAYS-2 randomized clinical trial. JAMA - J Am Med Assoc. 2018;320(8):779–791. 10.1001/jama.2018.11597 PMC6142999 30167701

[4] Hasegawa K , Hiraide A , Chang Y , Brown DFM. Association of prehospital advanced airway management with neurologic outcome and survival in patients with out-of-hospital cardiac arrest. JAMA - J Am Med Assoc. 2013;309(3):257–266. 10.1001/jama.2012.187612 23321764

[5] Loiselle JM , Cone DC. Effect of out-of-hospital pediatric endotracheal intubation on survival and neurological outcome: a controlled trial. Ann Emerg Med. 2001;38(3):352–353. 11548734

[6] Hanif MA , Kaji AH , Niemann JT. Advanced airway management does not improve outcome of out-of-hospital cardiac arrest. Acad Emerg Med. 2010;17(9):926–931. 20836772 10.1111/j.1553-2712.2010.00829.x

[7] Fouche PF , Simpson PM , Bendall J , Thomas RE , Cone DC , Doi SAR. Airways in out-of-hospital cardiac arrest: systematic review and meta-analysis. Prehosp Emerg Care. 2014;18(2):244–256. 24111481 10.3109/10903127.2013.831509

[8] Gausche M , Lewis RJ , Stratton SJ , et al. Effect of out-of-hospital pediatric endotracheal intubation on survival and neurological outcome: a controlled clinical trial. J Am Med Assoc. 2000;283(6):783–790. 10.1001/jama.283.6.783 10683058

[9] Lupton JR , Schmicker RH , Stephens S , et al. Outcomes with the use of bag–valve–mask ventilation during out-of-hospital cardiac arrest in the pragmatic airway resuscitation trial. Acad Emerg Med. 2020;27(5):366–374. 32220129 10.1111/acem.13927

[10] Ostermayer DG , Gausche-Hill M. Supraglottic airways: the history and current state of prehospital airway adjuncts. Prehosp Emerg Care. 2014;18(1):106–115. 24028649 10.3109/10903127.2013.825351

[11] Kurola J , Harve H , Kettunen T , et al. Airway management in cardiac arrest - comparison of the laryngeal tube, tracheal intubation and bag-valve mask ventilation in emergency medical training. Resuscitation. 2004;61(2):149–153. 15135191 10.1016/j.resuscitation.2004.01.014

[12] Braude D , Dixon D , Torres M , Martinez JP , O’Brien S , Bajema T. Brief research report: prehospital rapid sequence airway. Prehosp Emerg Care. 2021;25(4):583–587. 32628568 10.1080/10903127.2020.1792015

[13] Ruetzler K , Roessler B , Potura L , et al. Performance and skill retention of intubation by paramedics using seven different airway devices-a manikin study. Resuscitation. 2011;82(5):593–597. 21353364 10.1016/j.resuscitation.2011.01.008

[14] Merelman AH , Perlmutter MC , Strayer RJ. Alternatives to rapid sequence intubation: contemporary airway management with ketamine. West J Emerg Med. 2019;20(3):466–471. 31123547 10.5811/westjem.2019.4.42753 PMC6526883

[15] Weingart SD , Seth Trueger N , Wong N , Scofi J , Singh N , Rudolph SS. Delayed sequence intubation: a prospective observational study. Ann Emerg Med. 2015;65(4):349–355. 25447559 10.1016/j.annemergmed.2014.09.025

[16] Braude D , Richards M. Rapid Sequence Airway (RSA) - a novel approach to prehospital airway management. Prehosp Emerg Care. 2007;11(2):250–252. 17454819 10.1080/10903120701206032

[17] Colbert SA , OHanlon DM, Flanagan F, Page R, Moriarty DC. The laryngeal mask airway reduces blood flow in the common carotid artery bulb. Can J Anaesth. 1998;45(1):23–27. 9466022 10.1007/BF03011987

[18] Duckett J , Fell P , Han K , Kimber C , Taylor C. Introduction of the i-gel supraglottic airway device for prehospital airway management in a UK ambulance service. Emerg Med J. 2014;31(6):505–507. 23576232 10.1136/emermed-2012-202126

[19] Kim JG , Kim W , Kang GH , et al. Pre-hospital i-gel blind intubation for trauma: a simulation study. Clin Exp Emerg Med. 2018;5(1):29–34. 29618190 10.15441/ceem.16.188 PMC5891743

[20] Häske D , Schempf B , Gaier G , Niederberger C. Performance of the i-gel during pre-hospital cardiopulmonary resuscitation. Resuscitation. 2013;84(9):1229–1232. 23648215 10.1016/j.resuscitation.2013.04.025

[21] i-gel User Guide: Issue 4. Creative Techniques in Product and Engineering Design. 1991. https://www.intersurgical.com/content/files/80023/1103318462. Accessed July 11, 2021.

[22] Steuerwald MT , Braude DA , Petersen TR , Peterson K , Torres MA. Preliminary report: comparing aspiration rates between prehospital patients managed with extra glottic airway devices and endotracheal intubation. Air Med J. 2018;37(4):240–243. 29935702 10.1016/j.amj.2018.04.004

[23] Carney N , Totten AM , Cheney T , et al. Prehospital airway management: a systematic review. Prehosp Emerg Care. 2021;1–28. 10.1080/10903127.2021.1940400 34115570

